# Difficulties in Translating Appetite Sensations Effect of Turmeric-Based Beverage When Given Prior to Isoenergetic Medium- or High-Fat Meals in Healthy Subjects

**DOI:** 10.3390/nu11040736

**Published:** 2019-03-29

**Authors:** Yoghatama Cindya Zanzer, Ângela Giovana Batista, Anestis Dougkas, Juscelino Tovar, Yvonne Granfeldt, Elin Östman

**Affiliations:** 1 Food for Health Science Centre, Lund University, P.O. Box 124, SE-22100 Lund, Sweden; angelagiovanab@yahoo.com.br (Â.G.B.); anestis.dougkas@institutpaulbocuse.com (A.D.); juscelino.tovar@food.lth.se (J.T.); elin.ostman@goodideadrinks.com (E.Ö.); 2 Department of Food Technology, Engineering and Nutrition, Lund University, P.O. Box 124, SE-22100 Lund, Sweden; yvonne.granfeldt@food.lth.se; 3 School of Food Engineering, University of Campinas (UNICAMP), P.O. Box 6121, Campinas 13083-862, SP, Brazil; 4 Department of Food and Nutrition, Federal University of Santa Maria (UFSM), Campus Palmeira das Missões, Palmeira das Missões 98300-000, RS, Brazil; 5 Institute Paul Bocuse Research Centre, CEDEX, 69131 Ecully, France; 6 Good Idea Inc., Larkspur, CA 94939, USA

**Keywords:** turmeric, appetite, postprandial, isoenergetic, high fat, medium fat

## Abstract

The established effect of turmeric and its curcuminoids on appetite sensations was previously shown to be mediated by gut hormones release. In in vitro and preclinical studies, curcumin was shown to induce GLP-1 secretion and improve postprandial glycemia. In humans, consumption of 220 mL turmeric-based beverage (TUR, containing 185 mg gallic acid equivalents (GAE)) prior to white wheat bread (WWB, 50 g available carbohydrate) reduced early postprandial glucose levels and induced peptide tyrosine–tyrosine (PYY) release, as well as lowered ‘desire to eat’ and ‘prospective consumption’ in a postprandial setting, compared to control. In the present study, 12 healthy participants (5 men, 7 women) were admitted. An identical beverage was given and consumed prior to isoenergetic (423 kcal) medium-fat (MF) or high-fat (HF) meals. Appetite sensations including perceived ‘hunger’, ‘desire to eat’, ‘satiety’, ‘fullness’, ‘prospective consumption’, and ‘thirst’ were measured using visual analogue scales. MF induced 18% (*p* = 0.039) higher ‘satiety’ compared to HF. TUR consumption prior to either MF or HF did not modulate the perceived appetite sensations. Whether macronutrient-induced appetite sensations override the actual turmeric effects warrants further investigation.

## 1. Introduction

Obesity is characterized by a chronic imbalance between increased energy intake and decreased energy expenditure fostering the progression of other metabolic associated diseases such as type 2 diabetes (T2D) and ultimately cardiovascular disease (CVD). Given the close link between energy intake and appetite regulation, a better understanding of appetite control is essential to combat obesity. Several nutrients and food groups, including plants, which are rich in diverse bioactive compounds have received great attention as potential modulators of appetite [1,2]. However, little is known with regards to the impact of spices such as turmeric [3], black pepper [4], cinnamon [3,5,6,7], and ginger [8] on the regulation of appetite and appetite-related hormones such as peptide tyrosine–tyrosine (PYY), glucagon-like peptide 1 (GLP-1), and cholecystokinin (CCK). The release of PYY and GLP-1 induced by meals is known to be associated with the appetite suppression and delayed gastric emptying, among others. PYY could affect appetite via both direct central effect and gut motility which acts as an ileal brake, leading to a sensation of fullness and satiety [9]. Peripheral administration of GLP-1 has been shown to reduce appetite and *ad libitum* energy intake in humans [10]. Recent findings indicated that curcumin, a main compound of turmeric, has the ability to stimulate the secretion of GLP-1 from GLUTag cells, a model for the study of the GLP-1-secreting enteroendocrine L-cells [11]. To confirm those findings, Kato et al. performed an animal study by administrating curcumin (1.5 mg·kg^−1^) to rats [12]. Briefly, rats were preadministered with curcumin and 30 min later intraperitoneal glucose injections (2 g·kg^−1^) followed [12]. They showed that curcumin administration significantly increased GLP-1 and insulin levels after intraperitoneal glucose injections [12].

In a previous meal study, we showed that a turmeric-based beverage preload (TUR) significantly reduced early incremental blood glucose levels and lowered ‘desire to eat’ and ‘prospective consumption’, as well as increased PYY levels to a greater extent compared to control when consumed prior to a white wheat bread (WWB, containing 50 g available carbohydrate) meal challenge [3]. The present study set out to investigate if the changes in appetite are found also if TUR is consumed prior to breakfast meals varying in carbohydrate-to-fat ratio.

## 2. Ethical Aspect, Participants, and Methods

The study was approved by the Regional Ethical Review Board in Lund with identification number 2015/207 and registered in *clinicaltrials.gov* as NCT02479334. The study was randomized cross-over with at least one week wash-out period between the treatments. The randomization was carried out with permuted block design with a block size of four. The inclusion criteria of the study covered healthy subjects aged 18–55 years with body mass index (BMI) 20–28 kg·m^−2^ and having normal range of hemoglobin (men: 134–170 g·L^−1^, women: 117–153 g·L^−1^), creatinine (men: 60–105 µmol·L^−1^, women: 45–90 µmol·L^−1^), fasting triacylglycerol (TG) (0.4–1.7 mmol·L^−1^), and fasting blood glucose (3.5–5.5 mmol·L^−1^). Persons with any of the following were excluded: smoking or chewing tobacco, vegetarian or vegan diet, stressed by venous blood sampling or previous experience of cannulation difficulties, taking dietary supplement or receiving any drug treatment including use of nonsteroidal anti-inflammatory drugs such as asthma and allergy medications, pregnancy or breastfeeding, diagnosed with liver disease or diabetes mellitus, having food allergies and/or gastrointestinal disorder. In addition, women should be in the ovulation period of menstrual cycle (day 11–18) on the meal trial days. Written informed consent was obtained from all participants for the purpose of the screening process and meal study intervention. Participants were required to adhere to a low-phenolic diet 48 h prior to the experimental days. The list of food items permitted during the low-phenolic diet was provided by the investigator. In the morning after ~10 h overnight fasting, participants arrived in the clinical study facility and were allowed to take 10 min supine rest prior to cannulation and start of meal intervention. Briefly, participants received and consumed within 5 min the TUR beverage, and 10 min later a medium-fat (MF) or high-fat (HF) challenge meal was requested to be consumed within 10 min. Time counting started when participants took their first bite of the challenge meal.

**Figure 1:**
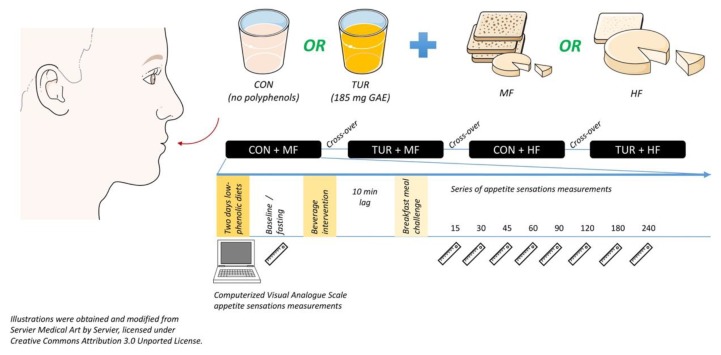
A schematic representation of the study design.

Similar to our previous study, the TUR beverage contained 185 mg GAE and was prepared as previously described [3], while the control beverage did not contain any GAE but had similar volume (220 mL) and nutritional composition (4 kJ/100 mL). Macronutrient composition of MF was 49.5 g or 46.7%E carbohydrate (CHO), 18.1 g or 38.5%E fat (F), and 15.7 g or 14.8%E protein (P), and the corresponding amount for HF were 12.4 g or 11.7%E CHO, 35.7 g or 76%E F, and 13 g or 12.3%E P. The MF consisted of white bread, 105 g; gouda cheese, 30 g; butter, 5 g; cream cheese, 5 g, and HF consisted of white bread, 25 g; gouda cheese, 42 g; butter, 20 g; cream cheese, 19 g. In this study, appetite sensations were included and measured electronically in provided laptops with the use of a 100 mm visual analogue scale (VAS) [13]. Appetite profiles, including ratings of ‘hunger’ (How hungry do you feel?), ‘desire to eat’ (How strong is your desire to eat?), ‘satiety’ (How satiated [i.e., pleasantly satisfied] are you?), ‘fullness’ (How full do you feel?), ‘prospective consumption’ (How much food do you think you could eat right now?), and ‘thirst‘ (How thirsty do you feel?) anchored by terms ‘not at all’ and ‘extremely’, were measured [14]. A schematic representation of the study design is depicted in Figure 1.

To evaluate the main effect of treatment, time, and treatment × time interaction, a mixed-model analysis using PROC-MIXED procedure (SAS Institute Inc., Cary, NC, USA) with repeated measures and an autoregressive covariance structure, followed by Tukey–Kramer *post hoc* test, was performed. The restricted maximum likelihood estimation (REML) with a Kenward–Roger correction was used in the model to adjust for small sample size bias. In the mixed-model analysis, participant was treated as random effect, time was included in the repeated effects, and statistical significance was set at *p-value* < 0.05. Data obtained are presented as the least square mean (LSM) ± standard error of the mean (SEM).

## 3. Results and Discussion

In the present study, 12 healthy participants (5 men, 7 women) were admitted to and successfully completed the study. Baseline characteristics of participants at the screening visit are presented in Table 1.

**Figure 2:**
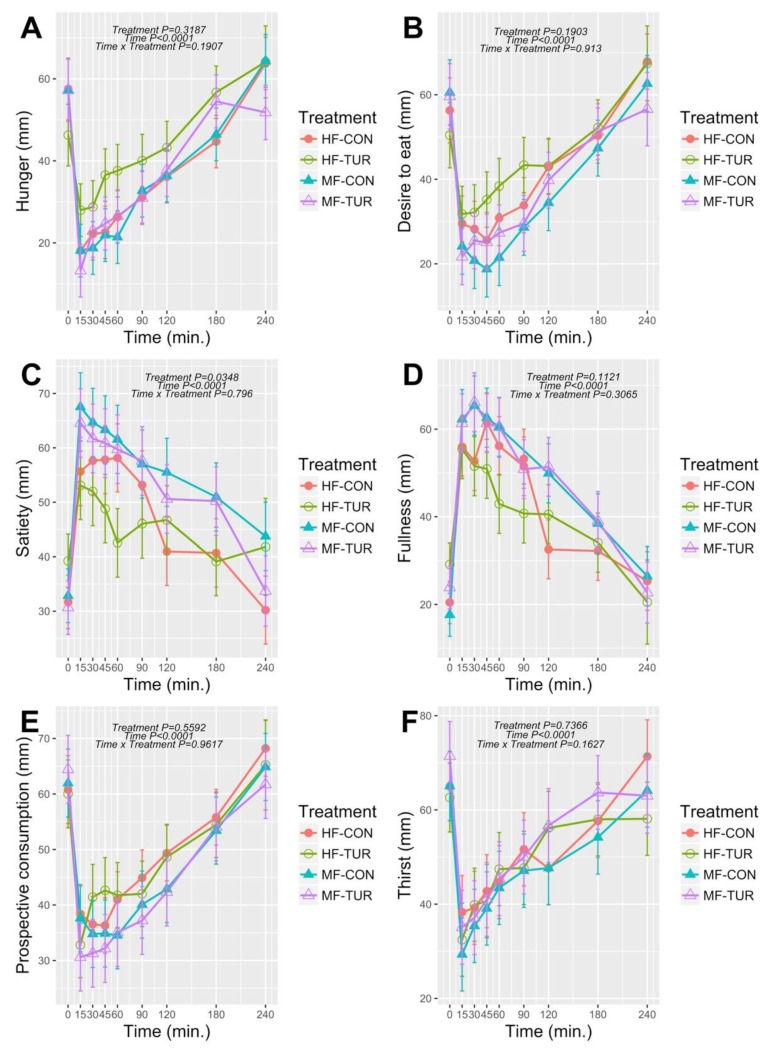
Postprandial response in ‘hunger’ (**A**), ‘desire to eat’ (**B**), ‘satiety’ (**C**), ‘fullness’ (**D**), ‘prospective consumption’ (**E**), and ‘thirst’ (**F**) after either control beverage + medium fat (MF), turmeric-based beverage + medium fat (MF-TUR), control beverage + high fat (HF), turmeric-based beverage + high fat (HF-TUR) in healthy subjects (LSMs ± SEM), *n* = 12.

There was only a main effect of treatment in perceived ‘satiety’ (*p* = 0.035), but not in other appetite sensations such as ‘hunger’ (*p* = 0.32), ‘desire to eat’ (*p* = 0.19), ‘fullness’ (*p* = 0.11), ‘prospective consumption’ (*p* = 0.56), and ‘thirst’ (*p* = 0.74) (Figure 2). No treatment × time interactions (*p* > 0.05) were observed for all appetite sensations. The results show that MF significantly induced 18% (*p* = 0.039) higher perceived ‘satiety’ compared to HF. The TUR, consumed prior to either MF or HF, did not modulate the perceived ‘satiety’ when compared to the control beverage consumed prior to MF or HF. The higher satiating effect elicited by the MF compared to HF meal is in agreement with other studies, which showed a higher satiating capacity of carbohydrate-rich relative to fat-rich meals [15,16,17]. As opposed to our previous study using WWB as a meal challenge [3], in the present study TUR did not modulate any appetite sensations after intake of either HF or MF. Although the MF contained the same amount of carbohydrate as the WWB used in our first study, the total energy of the MF was 160% higher compared to the WWB [3]. One could speculate that in such a complex meal matrix consisting of different types of food items varying in physical form, texture, and macronutrient composition, perceived satiety might be attributable to the macronutrient rather than the bioactive compounds content. If so, the elicited ‘satiety’ sensation could be partly mediated by gastric-emptying through ileal brake activation [18]. Although the role of different macronutrients in appetite regulation is not completely known yet, it depends partly on the food texture (i.e., liquid, semiliquid, solid, or mixed [19,20]). In a similar meal challenge using mixed texture food items, Marciani et al. observed that gastric emptying rate was significantly lower after a high-carbohydrate meal compared to high-fat meal in postprandial setting [21]. An earlier study showed that carbohydrates (starch/glucose blend) perfused into distal ileum decreased gastric emptying rate and induced PYY excretion [22]. An inverse correlation between gastric emptying and the amount of carbohydrate in the ileum was also observed in another study with healthy subjects [23]. Moreover, duodenal infusion of glucose has been shown to suppress hunger and reduce subsequent meal size in healthy men [24]. However, it should be kept in mind that the 185 mg GAE dose in TUR *per se* might not have been sufficient to elicit appetite-associated effects under the present meal challenge conditions. In this sense, further research on a dose–response effect and eventual toxicity seems prudent.

Some limitations such as lack of *ad libitum* food intake or gut-related hormone measurements exist in the present investigation. Although gut hormones are altered postprandially with a proven role in the digestion, absorption, and subsequent metabolic fate of nutrients, their physiological role in the regulation of eating behavior is still not fully understood [25]. Indeed, we recently showed that a black-pepper-based beverage can modulate overall appetite sensations without altering gut hormones (PYY and GLP-1) release [4]. In that report, it was speculated that other putative pathways such as activation of transient receptor potential (TRP) cation channels, which was previously shown to be activated by pungent spices such as cinnamon [26], might be involved in the modulation of appetite sensations. Although gender could potentially influence appetite ratings with some evidence showing women having higher ratings of fullness than men possibly due to differences in response to food cues, external influences (e.g., social, cultural norms), and sex hormones [27,28], inclusion of both sexes did not induce significant variation in the appetite ratings in this study.

## 4. Conclusions

Consumption of TUR prior to either MF or HF meals did not alter postmeal appetite sensations. Yet, ingestion of the control beverage prior to the MF meal induced significantly higher perceived ‘satiety’ compared to the HF meal challenge. However, the latter showed a main effect of treatment only, but no treatment × time interaction. The macronutrient composition and possibly other factors related to the meal challenge might confound appetite-related effects elicited by TUR. Future investigations evaluating the effect of plant-based bioactive compounds on appetite sensations should be performed with meal challenges varying in macronutrient composition.

## Figures and Tables

**Table 1:** Baseline characteristics of participants.

	Mean ± SEM
Age, y	26.5 ± 1.1
BMI, kg·m^−2^	23.8 ± 0.6
BMR, kcal	1615 ± 109
Body fat, %	24 ± 2.4
Blood glucose, mmol·L^−1^	4.98 ± 0.09
Insulin, pmol·L^−1^	67.14 ± 9.3
Triacylglycerol, mmol·L^−1^	0.85 ± 0.11
Total cholesterol, mmol·L^−1^	4.39 ± 0.26
Hb, g·L^−1^	146.7 ± 4.7
Creatinine, µmol·L^−1^	77.3 ± 3.4
ASAT, µkat·L^−1^	0.38 ± 0.03
ALAT, µkat·L^−1^	0.38 ± 0.07

Abbreviations: ALAT, alanine aminotransferase; ASAT, aspartate aminotransferase; BMI, body mass index; BMR, basal metabolic rate; Hb, hemoglobin; SEM, standard error of the mean.
